# Cellular and Molecular Gradients in the Ventral Horns With Increasing Distance From the Injury Site After Spinal Cord Contusion

**DOI:** 10.3389/fncel.2022.817752

**Published:** 2022-02-10

**Authors:** Ilyas M. Kabdesh, Yana O. Mukhamedshina, Svetlana S. Arkhipova, Davran K. Sabirov, Maxim S. Kuznecov, Alexandra B. Vyshtakalyuk, Albert A. Rizvanov, Victoria James, Yuri A. Chelyshev

**Affiliations:** ^1^OpenLab Gene and Cell Technologies, Institute of Fundamental Medicine and Biology, Kazan (Volga Region) Federal University, Kazan, Russia; ^2^Department of Histology, Cytology and Embryology, Kazan State Medical University, Kazan, Russia; ^3^Department of Epidemiology and Evidence Based Medicine, Kazan State Medical University, Kazan, Russia; ^4^FRC Kazan Scientific Center of RAS, A.E. Arbuzov Institute of Organic and Physical Chemistry, Kazan, Russia; ^5^Department of Zoology and General Biology, Institute of Fundamental Medicine and Biology, Kazan (Volga Region) Federal University, Kazan, Russia; ^6^Biodiscovery Institute, School of Veterinary Medicine and Science, University of Nottingham, Nottingham, United Kingdom

**Keywords:** NG2, astrocyte, axon-associated proteins, spinal cord injury, ventral horns

## Abstract

To identify cellular and molecular gradients following spinal cord injury (SCI), a rat contusion model of severe SCI was used to investigate the expression of NG2 and molecules that identify astrocytes and axons of the ventral horns (VH) at different distances on 7 and 30 days post-injury (dpi). A gradient of expression of NG2^+^/Olig2^+^ cells was determined, with the highest concentrations focused close to the injury site. A decrease in NG2 mean intensity correlates with a decrease in the number of NG2^+^ cells more distally. Immunoelectron microscopy subsequently revealed the presence of NG2 in connection with the membrane and within the cytoplasm of NG2^+^ glial cells and in large amounts within myelin membranes. Analysis of the astrocyte marker GFAP showed increased expression local to injury site from 7 dpi, this increase in expression spread more distally from the injury site by 30 dpi. Paradoxically, astrocyte perisynaptic processes marker GLT-1 was only increased in expression in areas remote from the epicenter, which was traced both at 7 and 30 dpi. Confocal microscopy showed a significant decrease in the number of 5-HT^+^ axons at a distance from the epicenter in the caudal direction, which is consistent with a decrease in β3-tubulin in these areas. The results indicate significant cellular and molecular reactions not only in the area of the gray matter damage but also in adjacent and remote areas, which is important for assessing the possibility of long-distance axonal growth.

## Introduction

Research that studies the cellular and molecular mechanisms in spinal cord injury has mainly focused on analyzing the injury area. After primary damage of the spinal cord, remote secondary damage usually occurs. A clinical neurophysiological and MRI studies indicated the development of these events in distant areas of patients’ spinal cord after spinal cord injury. Distinct spatiotemporal dynamics of tissue-specific neurodegeneration were found above and below spinal cord injury (David et al., 2021). Spatiotemporal experimental analysis of the gray and white matter of the spinal cord at a distance from the epicenter of injury shows the differential pattern and severity of pathological reactions, gene expression, and molecular regulation in the remote rostral and caudal regions (Ek et al., 2010; Yu et al., 2019). However, the main cause and mechanism of distant reactions, in contrast to shifts in the epicenter of damage, remain poorly understood. Meanwhile, this effect in distant regions seems to be important to take into account to assess the prospects for outcomes in pathology.

The functional deficit of neural networks in spinal cord injury (SCI) is a consequence of poor regeneration of damaged axons and insufficient target reinnervation. Axonal growth is inhibited by numerous inhibitory molecules of the extracellular matrix, such as chondroitin sulfate proteoglycans (CSPGs) (Ohtake and Li, 2015), as well as molecules associated with oligodendrocytes and myelin (Geoffroy and Zheng, 2014; Baldwin and Giger, 2015; McKerracher and Rosen, 2015). Under the influence of these inhibitors, dystrophic axons in the damaged area terminate growth and undergo retraction (Ramón y Cajal, 1928).

The idea that the expression level of neuron-glial antigen 2 (NG2) proteoglycan, also known as CSPG4, is elevated in the segments surrounding the SCI site in rodents is not new (Lemons et al., 1999; McTigue et al., 2006; Iaci et al., 2007). However, a detailed comparison of the expression levels and distribution of NG2 proteoglycan has not been performed in contusion SCI models. As mechanistic studies within these models begin to identify signaling pathways caused by the activation of CSPGs (Monnier et al., 2003; Duan and Giger, 2010; Coles et al., 2011), it becomes increasingly important to fully characterize the composition and distribution of CSPGs within the boundaries of scar formation.

In SCI, the area of damage contains numerous NG2^+^ glial cells (NG2 oligodendrocyte precursor cells, NG2/OPC) (Jones et al., 2002; Anderson et al., 2016) and is encased by a compact layer of reactive astrocytes that form a glial scar (Cregg et al., 2014; Hesp et al., 2018) hypothesize that formation of the glial scar depends on proliferating NG2^+^ cells, which include not only glia but also pericytes. They categorized NG2^+^ cells as glia or pericytes based on branched versus crescent-shaped morphology, respectively. Dividing NG2^+^ glia outnumber dividing NG2^+^ pericytes up to 30-fold, but are restricted to the glial scar and spared tissue, whereas dividing NG2^+^ pericytes enter lesions concomitant with angiogenesis (Hesp et al., 2018).

NG2^+^ cells rapidly proliferate in the area of damage, fix the axon growth cones on their surface, and prevent the dieback also known as axonal retraction of dystrophic axons, but at the same time inhibit their further growth (Filous and Schwab, 2018). This phenomenon is consistent with numerous data on the formation of synapse-like structures (synaptoid) “neuron – NG2^+^ cell” by NG2^+^ cells (Paukert and Bergles, 2006; Sakry et al., 2011; Kula et al., 2019). There is no unambiguous opinion about the influence of such structures on axon regeneration (Chelyshev et al., 2020). However, there are indications that NG2^+^ cells may enhance, facilitate, and support axon regeneration (de Castro et al., 2005; McTigue et al., 2006; Yang et al., 2006), as opposed to only influencing (Hossain-Ibrahim et al., 2007) or inhibiting growth (Dou and Levine, 1994; Tan et al., 2005, 2006; Donnelly et al., 2010).

Previous research has focused on how the state of cells and the extracellular matrix in the glial scar and the area immediately adjacent act to inhibit axonal growth. However, to provide extended axon growth and find targets far removed from the damaged area, it is important to characterize the expression of potential inhibitor molecules in morphologically intact tissue adjacent to the injury. Typical signs of degeneration are poorly manifested or completely absent in it, such as cell death, reorganization of the extracellular matrix, a decrease in tissue preservation, and pathological cavities. We wish to ascertain how the expression phenotype of glial cells changes, and if molecular shifts in the extracellular matrix occur in the areas potentially affecting extended axonal growth and synaptic condition.

There is evidence that glial cells located at a distance from the area of SCI, are involved in the pathological changes observed in neural networks. Activation of microglia and pro-inflammatory cytokines at a distance from the injury site predict the onset and severity of neuropathic pain after SCI (Zhao et al., 2007; Detloff et al., 2008; Gwak and Hulsebosch, 2009; Gwak et al., 2017). In this regard, data on the contributions of other types of glial cells, such as astrocytes and NG2^+^ cells, are still unclear. An increase in reactive astrogliosis and CSPGs, inhibitors of axon growth and plasticity, have been reported at sites distant from the lesion after severe mid-thoracic spinal contusion (Andrews et al., 2012). However, little is known about the signals that trigger such a remote cellular response.

Taking into account the special role of NG2^+^ glia in the control of neuronal plasticity, it would appear important to assess the reaction of these cells, as well as changes in the expression of NG2 proteoglycan in the area remote from the injury site, to determine the potential of extended axon growth. In this regard, we studied the spatial and temporal changes in the number of NG2 proteoglycan expressing cells, primarily NG2^+^ glia, as well as the expression of NG2 proteoglycan and markers of astrocytes and axons in a distance from the SCI epicenter in rat spinal cord contusion injury model.

## Materials and Methods

### Animals

Adult female Wistar rats (250–300 g, *n* = 45) were used for all experiments. Animals were randomly allocated to intact control (*n* = 15) and experimental (*n* = 30) groups. All animal protocols were approved by the Kazan Federal University Animal Care and Use Committee (No 2, May 5, 2015). Rats were housed under standard conditions (12 h light/dark cycle) with food and water available *ad libitum*.

### Surgery

After intramuscular injection of Zoletil (20 mg/kg, Virbac Sante Animale) experimental rats were deeply anesthetized under general anesthesia with isofluran. The fascia and paraspinous muscles were incised and after that laminectomy was performed at the Th8 vertebral level. SCI was induced using Infinite Horizon Impactor (Precision Systems and Instrumentation, LLC) and appropriate software (PSI IH Spinal Cord Impactor, version 5.0.3). The force of contusion was 300 kdyn (severe SCI; *n* = 30). After SCI gentamicin (25 mg/kg, Microgen) was injected intramuscularly once per day for 7 consecutive days post-injury (dpi). The urinary bladders were manually emptied twice each day until voiding commenced.

### Tissue Processing

Intact control (*n* = 5) and experimental rats at 7 and 30 dpi (*n* = 5 rats at each time-point; 7 and 30 dpi) were anesthetized and subjected to intracardiac perfusion with cold (4°C) 4% buffered formalin (BioVitrum). After perfusion, 30 mm segment centered around the injury site/Th8 was carefully isolated from the vertebral column, then fixed again in the 4% buffered formalin overnight. Distances 3–5, 6–8, and 10–12 mm from the visualized epicenter of the injury were determined on the isolated fragment of the spinal cord according to the data of our previous studies (Mukhamedshina et al., 2019). The next day, spinal cords were cryopreserved in a sucrose gradient (15 and 30%). For the analysis of the VH, we used longitudinal sections (20 μm thick) of the spinal cord in areas remote from the site of injury/Th8, obtained using a Tissue-Tek Cryo3 DM (Sakura) cryostat. The choice of VH as the object of research is based on the presence of key modules of the spinal locomotor circuit in this area.

### Transmission Electron Microscopy

For transmission electron microscopy, samples were fixed in 10% formaldehyde mixed with 0.2% phosphate-buffered glutaraldehyde solution (Alfa Aesar by Thermo Fisher Scientific, Germany) at 4°C for 12 h, and post-fixed in a 0.5% OsO_4_ (Sigma–Aldrich, United States) for 1 h. Following fixation, samples were dehydrated in an ethanol gradient and embedded in LR White (Electron Microscopy Sciences, Hatfield). The 0.1 μm ultrathin sections were mounted on Formvar-coated Ni grids. For Immune gold cytochemistry the sections were blocked with TBS-NDS-BSA-TX100 [Tris-buffered saline (Tris 0.01 M, NaCl 0.15 M pH = 8.2), normal donkey serum 10%, bovine serum albumin 0.2%, and Triton X-100 0.1%] for 1 h. After washing in TBS, the sections were incubated overnight at 4°C with anti-NG2 and anti-ALDH1L1 (aldehyde dehydrogenase 1 family member L1) (Table 1) antibodies (Abs) and then with secondary Abs conjugated to colloidal gold (5 nm particles to evaluate ALDH1L1 and 10 nm particles to evaluate NG2 proteoglycan) (Sigma–Aldrich, United States) for 2 h at room temperature (RT).

**TABLE 1:** Primary and secondary antibodies used in Western blotting (WB), immunohistochemistry (IHC), and immunoelectron microscopy (IEM) tests.

Antibody	Host	Dilution	Source
GFAP	mouse	1:1,000 (WB)	Santa Cruz (sc-33673)
GLT-1	rabbit	1:1,000 (WB)	Abcam (ab41621)
β-III-tubulin	mouse	1:500 (WB)	Santa Cruz (sc-5274)
NG2	mouse	1:100 (IHC) 1:100 (IEM)	Sigma–Aldrich (N8912)
ALDH1L1	rabbit	1:100 (IEM)	Abcam (ab87117)
5-HT	goat	1:400 (IHC)	Abcam (ab66047)
NeuN	rabbit	1:100 (IHC)	Sigma-Aldrich (SAB4300883)
Olig2	rabbit	1:75 (IHC)	Santa Cruz (sc-48817)
Anti-goat IgG conjugated to Alexa 488	donkey	1:200 (IHC)	Thermo Fisher (A11055)
Anti-mouse IgG conjugated to Alexa 555	donkey	1:200 (IHC)	Thermo Fisher (A31570)
Anti-rabbit IgG conjugated to Alexa 647	donkey	1:200 (IHC)	Thermo Fisher (A31573)
HRP-conjugated anti-rabbit IgG	goat	1:2,000 (WB)	Cell Signaling (7074P2)
HRP-conjugated anti-mouse IgG	goat	1:1,000 (WB)	Sigma–Aldrich (A4416)

Then sections were stained with uranyl acetate and lead citrate and evaluated using a transmission electron microscope (Hitachi HT7700, Tokyo, Japan). To measure the size of gold nanoparticles, as well as the thickness of the filaments, a ZEN blue Lite program and software supplied with the Hitachi 7700 transmission electron microscope were used.

### Immunohistochemistry

Longitudinal spinal cord sections were processed for immunohistochemistry as previously described (Mukhamedshina et al., 2019). Primary and fluorescence secondary Abs are described in Table 1. Nuclear stain *via* 4′,6-diamidino-2-phenylindole (DAPI) (10 μg/mL, Sigma). Coverslips were mounted on slides using ImmunoHistoMount medium (ab104131, Abcam). Sections incubated only with secondary Abs (without primary Abs) were used as a reaction control. The slides were examined and photographed using LSM 700 confocal microscopy (Carl Zeiss). Three-dimensional reconstruction and cell quantification/semi-quantitative analyses were performed with Zen 2012 software (Carl Zeiss).

### Cell Quantification

Five sections per rat in the VH area at distances of 3–5, 6–8, and 10–12 mm in the caudal direction were analyzed in intact control (*n* = 5) and experimental (*n* = 5 rats at each time-point; 7 and 30 dpi) groups. In the confocal images of this area, the arithmetic mean intensities of fluorescence (MIF units, semi-quantitative analysis) of NG2 proteoglycan was measured as previously described (Povysheva et al., 2018). In each VH area we examined 3 zones with *S* = 0.02 mm^2^, for each channel, the lowest intensity signals were removed to minimize the background. For semi-quantitative analysis, all sections were imaged using identical confocal settings (laser intensity, gain, offset).

For quantification analyses, 4 types of cells were selected and examined in *S* = 0.04 mm^2^: NG2^–^/Olig2^+^ cells, NG2^+^ pericyte with crescent morphology (Hesp et al., 2018), branched NG2^+^ oval shape cells, and NG2^+^/Olig2^+^ cells (double-positive). 5-Hydroxytryptamine (5-HT)^+^ axon profiles (>0.5 μm in length) were counted within VH area containing at least two neurons (NeuN were used for visualization neurons) as previously described (Freria et al., 2017). For 5-HT puncta analysis ImageJ software (version 1.52a, National Institutes of Health) and Puncta Analyzer v. 2.0 plug-in were used. The Z-stack (*S* = 0.01 mm^2^) was used for more reliable counting of the number of 5-HT^+^ axon profiles. Investigations were validated by two observers to ensure the correct identification of the immunoreactivity patterns. Both investigators were blinded to the experimental group.

### Western Blotting

The spinal cord for western blotting was isolated from intact controls (*n* = 5) and experimental rats (*n* = 5 rats at each timepoint; 7 and 30 dpi) and washed with sterile phosphate-buffered saline (PBS) twice. Using microdissection technique, including micromanipulations with fragments of the spinal cord, controlled through stereomicroscope with LED illumination (Carl Zeiss) and aimed at separating white and gray matter with subsequent isolation of VH using microinstrumentation, the VH (3–5, 6–8, and 10–12 mm caudally from the injury site/Th8) were isolated and stored at –80°C. For VH total protein isolation samples were dissolved in radioimmunoprecipitation assay (RIPA) lysis buffer (Cell Signaling Technology) with protease inhibitor cocktail (Thermo Fisher Scientific), then incubated on multi-rotator (Biosan) for 1 h at 4°C, homogenized *via* FastPrep-24 Classic bead beating grinder and lysis system (MP Biomedicals) at a speed 4 m/s for 20 s, sonicated in ultrasonic bath for 10 min and finally centrifuged at 15,000 rpm for 30 min at 4°C. Total protein concentrations were measured using the BCA Protein Assay Kit (Thermo Fisher Scientific). 2 × SDS (sodium dodecyl sulfate) sample buffer was added to each sample before incubating at 95°C for 5 min. A 5 μg of protein sample was loaded to each lane of 4–12% gradient SDS-PAGE gel and then transferred to a polyvinylidene fluoride (PVDF) membrane (Bio-Rad Laboratories) after electrophoresis. The PVDF membrane was blocked with 5% non-fat dry milk diluted in PBS with Tween-20 (PBS-T) (pH 7.4) for 1 h at RT. The primary Abs (Table 1) were diluted and incubated with membranes overnight at 4°C. The next day, after washing in PBS-T the membranes it was incubated with HRP-conjugated secondary Abs in 2 h at RT and visualized with the Clarity Western ECL Substrate kit (Bio-Rad Laboratories). Primary and secondary Abs were diluted in 5% non-fat dry milk in PBS-T. Analysis of the western blotting results with total protein normalization was performed using Image Lab software (Bio-Rad Laboratories).

### Statistical Analysis

Obtained data analysis was performed using R 3.6.3 software (R Foundation for Statistical Computing, Vienna, Austria). Descriptive statistics are presented as mean ± standard deviation (median, first, and third quartile). Sample distributions are visually represented as boxplots. Repeated measures for one animal were averaged. To analyze differences between samples, the Kruskal–Wallis test was used and Dunn’s test was implemented as *post hoc* test with Holm adjustment for multiple comparisons. Mann–Whitney *U* test, using Origin 8.0 software, was applied for the statistical analysis of Western blot data.

## Results

### Assessment of NG2 Proteoglycan Expressing Cells With Increasing Distance From the Injury Site in the Caudal Direction

**FIGURE 1:**
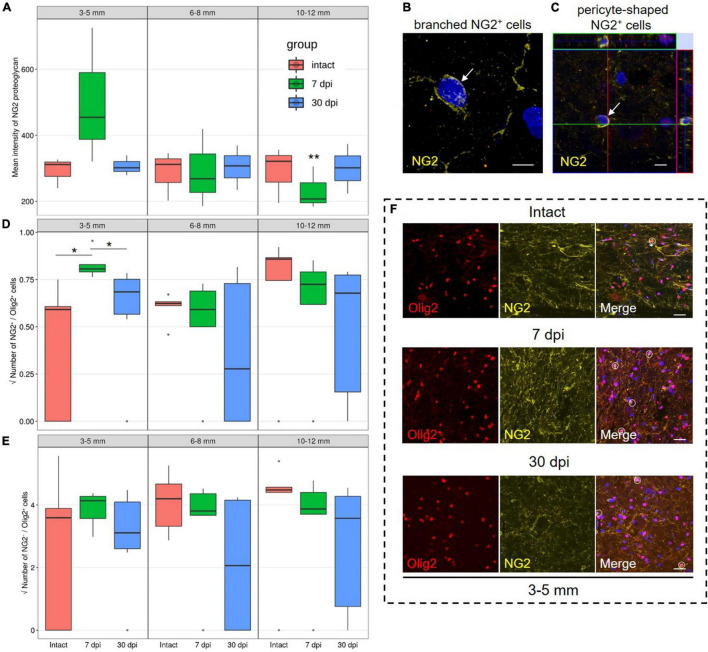
Assessment of NG2 expressing cells and NG2^–^/Olig2^+^ cells identification. **(A)** The mean intensity of NG2 (MIF units, *Y*-axis) in the intact spinal cord (red column), 7 (green column), and 30 (blue column) dpi in the ventral horns (VH) 3–5, 6–8, and 10–12 mm caudally from the injury epicenter. **P < 0.05 – compared with 7 dpi group and distance of 3–5 mm from the epicenter. Visualization of the branched-shaped **(B)** and pericyte-shaped **(C)** NG2^+^ cells (arrows). Three-dimensional confocal microscopy images are shown. Nuclei were counterstained with 4′,6-diamidino-2-phenylindole (DAPI). The square root of NG2^+^/Olig2^+^ **(D)** and NG2^–^/Olig2^+^ **(E)** cells number in the examined regions, *P < 0.05. **(F)** Visualization of the NG2^–^/Olig2^+^ (red channel) and NG2^+^/Olig2^+^ cells (merge channel, circle) in intact spinal cord, 7 and 30 dpi in VH, 3–5 mm caudally from the injury epicenter. Scale bar = 5 **(B,C)** and 20 **(F)** μm.

Evaluation of the mean fluorescence intensity of NG2 proteoglycan in the VH showed the highest expression by 7 dpi at a distance of 3–5 mm from the epicenter when compared with 10–12 mm distance at the same timepoint [454.5 (387.7–590.0) at 3–5 mm vs. 207.2 (195.6–256.4) at 10–12 mm, *P* < 0.05]. While expression changed across these distances, we did not find any significant differences between the intact control and experimental groups (Figure 1A).

We performed a quantitative analysis of three populations of NG2^+^ cells: NG2^+^/Olig2^+^, branched-shaped, and pericyte-shaped NG2^+^ cells. In the VH, with the distance from the SCI epicenter, the number of branched-shaped and pericyte-shaped NG2^+^ cells did not differ significantly. These cells may be absent, or only present in very small numbers not present in the fields of view analyzed (Figures 1B,C).

The population of NG2^+^/Olig2^+^ cells in the VH was also relatively small. However, at 7 dpi and a distance of 3–5 mm from the SCI epicenter, a larger number of these cells were found in comparison with intact controls and in the later 30 dpi timepoint [0.65 (0.62–0.69) at 7 dpi vs. 0.35 (0.00–0.37) at intact control and 0.47 (0.32–0.57) at 30 dpi, *P* < 0.05] (Figures 1D,F). At 7 dpi, the number of NG2^+^/Olig2^+^ cells decreased (*P* < 0.05) at a distance of 6–8 mm when compared with the area closer (3–5 mm) or more distant (10–12 mm) to the epicenter of the injury. At the same time, in the VH, the number of NG2^–^/Olig2^+^ cells not expressing NG2 proteoglycan was more than 15 times higher than the number of NG2^+^/Olig2^+^ cells in all studied groups in the corresponding zones (Figures 1E,F).

### Assessment of Astrocyte Markers

**FIGURE 2:**
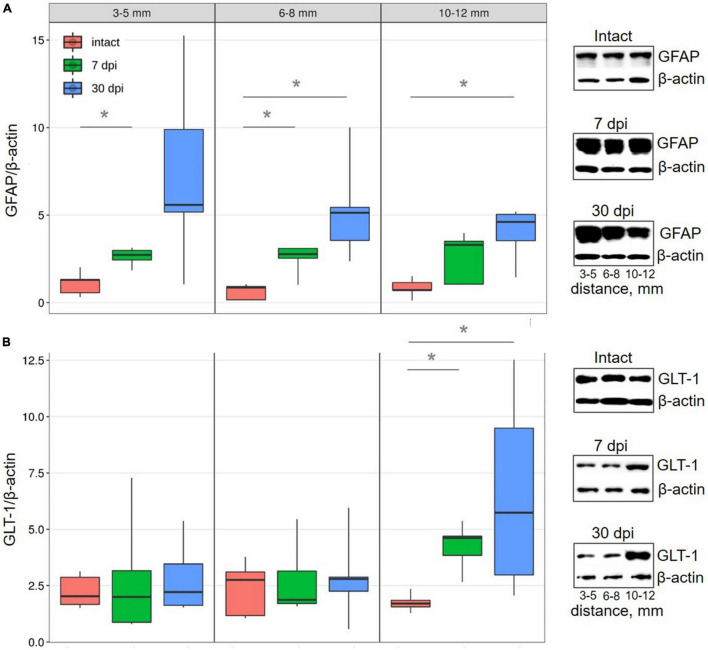
Western blot analysis of astrocytes markers in the VH with increasing distance from the injury site in the caudal direction. Representative images of western blots showing GFAP **(A)** and GLT-1 **(B)** and their corresponding control – β-actin. Data are presented as the ratio of the number of target proteins to the reference protein (β-actin), the average value (*n* = 5 rats per group). *P < 0.05, Mann–Whitney test.

Following analysis of all selected distances from the SCI epicenter, it was found that glial fibrillary acidic protein (GFAP) expression in the VH increased by ∼ 2-fold at 7 dpi, a trend that continued to 30 dpi (Figure 2A). These results indicate the development of reactive astrogliosis following a spinal cord injury across a wide area that spans a considerable distance from the epicenter of the injury. In addition, these data indicate a sufficient duration of reactive astrogliosis both near the epicenter and at a distance from it.

Conversely, there was no detectable change in the expression of glutamate transporter 1 (GLT-1), which is mainly localized in astrocytes and their perisynaptic processes, within regions close to (3–8 mm) the injury epicenter in all studied groups. However, more distally (10–12 mm) from the epicenter of damage, GLT-1 expression increased (∼2.4 times) by 7 dpi (*P* < 0.05) and increased (∼3.7 times) further by 30 dpi, when compared with intact controls (Figure 2B).

### Astrocyte and NG2 Proteoglycan Expressing Cells in Ventral Horns: Immunoelectron Microscopy

**FIGURE 3:**
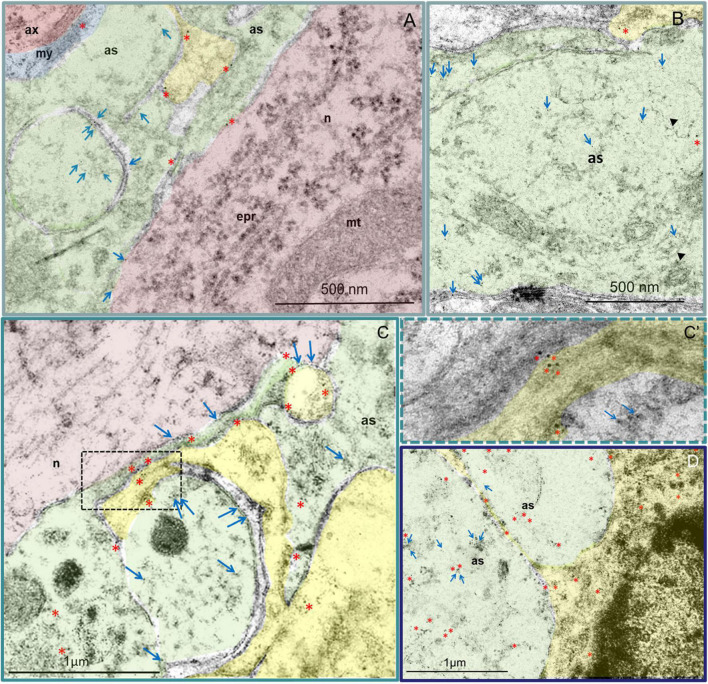
Astrocytes (green) and NG2^+^ (yellow) cells in the VH. Immunoelectron microscopic images of intact **(A,B)** and injured spinal cord 30 dpi 3–5 mm **(C)** and 6–8 mm **(D)** caudally from the epicenter. Asterisks denote 10 nm golden nanoparticles (anti-NG2 antibody) and arrows highlight 5 nm golden nanoparticles (anti-ALDH1L1 antibody). **(A)** In the VH of the intact spinal cord, a positive reaction to NG2 proteoglycan was detected in the cytoplasm of oligodendrocytes and myelin membranes, as well as in a small amount along the plasma membranes in the processes of astrocytes, including those adjacent to the neuron. In NG2^+^ glia, the ALDH1L1 immunopositive reaction was not observed. **(B)** High ALDH1L1 immunoreactivity was found in the cytoplasm of astrocytes. The belonging of cells to astrocytes can be estimated by the presence in the cytoplasm of characteristic lumps of average electron density and filaments 15 nm in diameter (head arrow). **(C)** At 30 dpi, the distributions of NG2 proteoglycan and ALDH1L1 were similar to those of the intact spinal cord. At the same time, the expression of NG2 proteoglycan in NG2^+^ glial cells, oligodendrocytes **(C)**, and reactive astrocytes **(D)** was visually more intense. Higher-magnification view of the dashed boxed area in the **(C′)**. as – astrocyte; ax – axon; epr – endoplasmic reticulum; mt – mitochondria; my – myelin; n – neuron. Scale bar: 500 nm **(A,B)** and 1 μm **(C,D)**.

In the VH of the intact spinal cord, NG2 proteoglycan was visible in the cytoplasm of oligodendrocytes and myelin membranes (Figure 3A). The perisynaptic region also contained another population of NG2^+^/ALDH1L1^–^ cells with thick and short processes and irregular cell bodies with polymorphic nuclei. Astrocytes surrounding neurons exhibited weak immunoreactivity of NG2 proteoglycan along the plasma membranes, while also demonstrating immunoreactivity of ALDH1L1, predominantly in the cytoplasm, as well as on the surface membrane of the processes (Figure 3A). Astrocytic morphology of these cells was confirmed by the presence of distinctive lumps of average electron density and intermediate filaments 15 nm in diameter (Figure 3B) in the cytoplasm.

In the spinal cord at 30 dpi, in areas remote from the epicenter of the injury in the caudal direction on longitudinal sections of the VH, three zones can be distinguished in the dorsoventral direction: the damaged degenerated substance, the area of the glial barrier (there is a strong interlacing of processes of glial and nerve cells, as intact and degenerating) and the area of intact tissue. At the distance farthest from the epicenter of the injury, there is no discernable zone of degenerated substance and the expression of NG2 proteoglycan and ALDH1L1 in the above-mentioned cells, as well as their morphology, was similar to the intact spinal cord. At the same time, the expression of NG2 proteoglycan in NG2^+^ glial cells, oligodendrocytes, and reactive astrocytes was visually more intense (Figures 3C,C’,D). In the zone of destruction and glial scar, activated astrocytes had characteristic (specific) ultrastructural features of the cytoplasm structure and more pronounced expression of NG2 proteoglycan and ALDH1L1 when compared with the intact control group or intact tissues far from the lesion epicenter (Figure 3C).

NG2^+^ cells and their processes were found in large numbers and intimately associated with reactive astrocytes. Whilst these cells were present at different distances from the epicenter, they were mainly in the area of the glial scar. Processes of reactive astrocytes with electron-transparent cytoplasm were found between the more electron-dense granular processes of NG2^+^ cells. In astrocytes processes, à mild immunopositive reaction to NG2 proteoglycan was found mainly on membranes, whilst an intense immunopositive reaction to ALDH1L1 in the cytoplasm was visualized (Figure 3D).

### Expression of Axon-Associated Proteins

**FIGURE 4:**
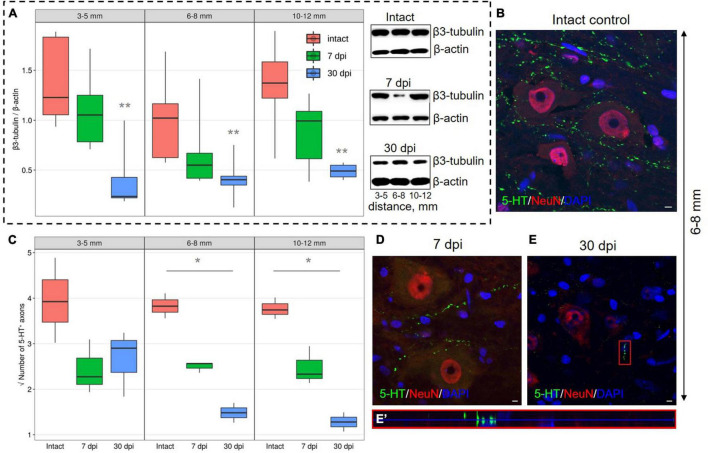
Estimation of axon-associated proteins in the ventral horns (VH) with increasing distance from the injury site in the caudal direction. **(A)** Western blot analysis of β3-tubulin, β-actin was used as a loading control. Data are presented as the ratio of the quantity of target protein (β3-tubulin) to the reference protein (β-actin), the average value (*n* = 5 rats per group). **P < 0.05 – compared with the intact group at an appropriate distance from the epicenter, Mann–Whitney *U* test. Visualization of 5-HT^+^ axons in the intact spinal cord **(B)**, 7 **(D)**, and 30 **(E)** dpi in VH 6–8 mm caudally from the injury epicenter. 5-HT^+^ axons at 30 dpi are marked with red boxed area and are shown enlarged in another X, Y, and Z positions in **(E′)**. Three-dimensional confocal microscopy images are shown. Nuclei were counterstained with 4′,6-diamidino-2-phenylindole (DAPI). **(C)** Square root of 5-HT^+^ axons (*Y*-axis) number in the intact spinal cord (red column), 7 (green column), and 30 (blue column) dpi in VH 3–5, 6–8, and 10–12 mm caudally from the injury epicenter, *P < 0.05.

Expression of β3-tubulin, to determine the microtubules of neurons in the VH showed decreased expression (*P* < 0.05) at a distance of 3–5, 6–8, and 10–12 mm from the injury epicenter by 30 dpi when compared with the intact control groups (Figure 4A). At 7 dpi, at all distances from the epicenter, β3-tubulin content also decreases, but the significance of these differences when compared with intact controls was not confirmed.

A significant decrease in the number of 5-HT^+^ axons in the VH by 30 dpi in comparison with intact control at distances of 6–8 and 10–12 mm from the epicenter of the injury was also found (Figures 4B–E′). By 30 dpi at a distance of 3–5 mm from the SCI epicenter, the amount of 5-HT^+^ axons in comparison with 7 dpi remained the same, but the number decreased at distances of 6–8 and 10–12 mm caudally for the same time points.

It should be noted that all our results are presented in this study only for areas in the caudal direction from the injury site.

## Discussion

In traumatic injury, axons degenerate not only in the caudal region but also proximal to the site of spinal cord injury (Freund et al., 2012). Proteomic analysis revealed differences in the spectrum of bioactive molecules when comparing the rostral and caudal regions in relation to the epicenter of injury, with a predominance of molecular markers of cell death in the caudal site (Cizkova et al., 2014). In spinal cord injury, pathological shifts in the caudal region lead to severe locomotor deficits. We focused on the analysis of the ventral horns in the perilesional area along the rostrocaudal axis because of the importance of cellular and molecular shifts in this region for the restoration of locomotor function.

In severe contusion SCI, an increase in the mean fluorescence intensity of NG2 proteoglycan in the VH at a distance of 3–5 mm from the epicenter at 7 dpi was confirmed, this is accompanied by a decrease in more distal regions (10–12 mm). Since the number of NG2^+^ cells does not change significantly at a distance of 10–12 mm, this effect can be associated with a decrease in the expression of NG2 proteoglycan in this remote zone. By 30 dpi, the expression of NG2 proteoglycan at all the studied distances from the epicenter does not differ from that in intact animals in the VH of the corresponding anatomical regions. The observed dynamics of expression of NG2 proteoglycan can be associated primarily with the population of NG2^+^ glial cells, although this proteoglycan is also expressed by cells of other types, such as pericytes (Hesp et al., 2018), meningeal fibroblasts, and macrophages (Bu et al., 2001; Jones et al., 2002). The observed increase in NG2 proteoglycan content at a term of 7 dpi in the area close to the lesion epicenter, may be associated with the well-known rapid increase in the amount of NG2^+^ glia and subsequent NG2 proteoglycan expression in this area (Filous and Schwab, 2018). This proteoglycan binds FGF2 in a glycosaminoglycan-independent, core protein-mediated manner (Cattaruzza et al., 2013), retaining this neurotrophic and angiogenic molecule on the cell membrane for subsequent receptor presentation.

In the VH with distance from the SCI epicenter, the dynamics of the populations of NG2^+^/Olig2^+^ cells and branched NG2^+^ cells differ, which may indirectly indicate their heterogeneity. To a greater extent, as it turned out, NG2^+^/Olig2^+^ cells respond to SCI in areas close to the damage epicenter, in contrast to NG2^–^/Olig2^+^ cells, the population of which, judging by our data, significantly exceeds the population of NG2^+^/Olig2^+^ cells. NG2^+^/Olig2^+^ cells react rapidly to SCI and this reaction is characterized by cell body swelling, retraction of cell processes and increased expression of NG2 proteoglycan (Levine, 2016; Hesp et al., 2018) showed that in mice NG2^+^/Olig2^+^ cells were observed in large numbers in the area of the glial scar, while absent in the area of the epicenter of the injury after SCI (Hesp et al., 2018).

It is assumed that when damaged in the central nervous system (CNS), NG2^+^ glia actively comes into contact with axons, inhibiting their retraction (Filous and Schwab, 2018). However, it remains unclear how long axons in which retraction is prevented and are thwarted by contact with NG2 cells can maintain their regenerative potential and continue to grow out to their targets (Chelyshev et al., 2020). It can also be assumed that thwarting axon growth as a result of their fixation on glial cell surfaces is important not only to prevent their dieback but also as a signal to start sprouting (Geoffroy and Zheng, 2014). NG2 proteoglycan has been shown to enhance serotonergic axon sprouting. It has been shown on NG2 proteoglycan knockouts in SCI that this proteoglycan enhances the penetration of serotonergic fibers into the scar tissue (de Castro et al., 2005). The number of 5-HT^+^ axons, according to our data, significantly decreases at distances farther from the SCI epicenter by 30 dpi, in contrast to areas close to the epicenter of the injury and when compared with the intact spinal cord. We found that this was not caused by the inhibitory effect of cells expressing proteoglycan NG2, since we did not find quantitative or qualitative changes in the populations of these cells at longer distances (6–8, 10–12 mm) from the SCI epicenter. However, at a distance of 3–5 mm caudal to the lesion area, the decrease in the number of serotonergic axons is consistent with an increase in the number of NG2^+^/Olig2^+^ cells, which may indicate the prevention of axon dieback by this cell population. It is assumed that the effect of NG2 proteoglycan on axonal growth is mediated by the activation of protein kinase C zeta (PKCζ), an atypical molecule for immobilizing dystrophic axons, and this activation is both necessary and sufficient to inhibit axonal growth (Lee et al., 2013). It has been suggested that the immobilization of dystrophic growth cones involves the PTPσ molecule, which, like LARs, but not NgRs, accumulates in the penumbra region during SCI (Lang et al., 2015).

We also found a decrease in the content of β3-tubulin to the same extent in all studied removal zones, which can be explained by a decrease in the number of nerve fibers due to their degeneration, a decrease in axonal transport in preserved fibers, or both of these processes. The observed dynamics of this axon marker in the VH may be associated with a decrease in its synthesis in neurons, inhibition of anterograde transport in preserved descending axons, or with a decrease in the number of the same axons.

The possibility of expression of NG2 proteoglycan by reactive astrocytes of the spinal cord has been shown earlier (Anderson et al., 2016). At the same time, using the method of electron microscopic immunocytochemistry, we clarified the possibility of NG2 expression in reactive astrocytes located near the epicenter of damage, and those astrocytes that are located at a distance from the injury area. It was found that NG2 proteoglycan expressing astrocytes make up approximately 25% of the total number of NG2^+^ cells in the glial scar by 4 weeks after SCI (Hackett et al., 2018). NG2^+^ cells also display wide differentiation potential and give rise to reactive astrocytes during ischemic lesion under the influence of Shh signaling activation (Honsa et al., 2016). We found that NG2 proteoglycan expression was visible not only in post-traumatic reactive astrocytes but also in astrocytes of the VH in the intact spinal cord. This observation is consistent with the idea that NG2^+^ cells can differentiate into GFAP^+^ astrocytes, which has been shown in several models of CNS injury (Sellers et al., 2009; Tripathi et al., 2010; Komitova et al., 2011).

Judging by the expression of GFAP, reactive astrogliosis at a period of 30 dpi is observed at a considerable distance from the epicenter of the contusion injury and does not grow in the caudal direction from a distance of 6–8 mm. In connection with astrocytes, we stated a paradoxical reaction of an increase in GLT-1 expression at the maximum studied distance at 30 dpi. The absence of shifts in this indicator in the zone close to the damaged area may be associated with the disintegration of synapses, and in the remote area, where synapses are preserved, astrocytes increase the expression of GLT-1 to maintain the functioning of glutamatergic synapses. The increase in GLT-1 that we found in the gray matter area remote from the epicenter may also be associated with the growth and branching of regenerating axons and their participation in the restoration of synaptic contacts, which is not observed near the lesion focus, where reactive astrocytes are present that are not involved in the formation of synapses. This observation is the first indication of differences in the reactions of astrocytes located near and far from the area of tissue damage and destruction.

## Data Availability Statement

The raw data supporting the conclusions of this article will be made available by the authors, without undue reservation.

## Ethics Statement

The animal study was reviewed and approved by the Kazan Federal University Animal Care and Use Committee (No 2, 5 May 2015).

## Author Contributions

YC and YM contributed to the conceptualization, methodology, and supervision. IK, DS, and SA contributed to the investigation. IK, DS, MK, YM, and SA contributed to the formal analysis. YC, AV, and AR contributed to obtaining the resources. YC, YM, and IK contributed to writing the original draft. YC, YM, IK, SA, and VJ contributed to writing, reviewing, and editing the manuscript. IK, MK, YM, and SA visualization. YC and AR contributed toward funding acquisition. All authors had full access to all the data in the study and take responsibility for the integrity of the data and the accuracy of the data analysis.

## Conflict of Interest

The authors declare that the research was conducted in the absence of any commercial or financial relationships that could be construed as a potential conflict of interest.

## Publisher’s Note

All claims expressed in this article are solely those of the authors and do not necessarily represent those of their affiliated organizations, or those of the publisher, the editors and the reviewers. Any product that may be evaluated in this article, or claim that may be made by its manufacturer, is not guaranteed or endorsed by the publisher.
